# Prohibited Grazing Policy Satisfaction and Life Satisfaction in Rural Northwest China—A Case Study in Yanchi County, Ningxia Hui Autonomous Region

**DOI:** 10.3390/ijerph16224374

**Published:** 2019-11-08

**Authors:** Weiwei Wang, Lihua Zhou, Guojing Yang, Yan Sun, Yong Chen

**Affiliations:** 1 Key Laboratory of Desert and Desertification, Northwest Institute of Eco-Environment and Resources, Chinese Academy of Sciences, Lanzhou 730000, China; wangweiyudong@163.com (W.W.); chenyong@lzb.ac.cn (Y.C.); 2 University of Chinese Academy of Sciences, Beijing 100049, China; 3 Institutes of Science and Development, Chinese Academy of Sciences, Beijing 100190, China; 4 Key Laboratory of Ecohydrology of Inland River Basin, Northwest Institute of Eco-Environment and Resources, Chinese Academy of Sciences, Lanzhou 730000, China; ygj7518@163.com; 5 College of Earth and Environmental Sciences, Lanzhou University, Lanzhou 730000, China; saadiya99@126.com

**Keywords:** life satisfaction, PGP satisfaction, grassland restoration, farmers, Yanchi County, China

## Abstract

In order to restore degraded grasslands, the Chinese central government initiated the Prohibited Grazing Policy (PGP) in areas of severe grassland degradation and ecologically fragile regions which is an important payment for ecosystem services (PES) program. Since the initiation of this policy in the early 2000s, the PGP has significantly influenced participants’ lives. Therefore, in order for the policy to be successful, it is necessary to understand what determines participants’ satisfaction in the policy. This paper presents an analysis of survey data from Yanchi County using ordered probit regression models to explore the factors influencing PGP satisfaction and life satisfaction. The empirical results suggest that farmers’ policy perception, environmental perception, and livelihood strategies of raising sheep had significant effects on PGP satisfaction. Additionally, PGP satisfaction, marital status, environmental satisfaction, self-reported influence of the PGP on income, self-reported income level, and self-reported income and expenditure had significantly positive effects on overall life satisfaction. These results are important for promoting better implementation of such programs as well as enhancing social stability and sustainable development in these regions.

## 1. Introduction

In the 1950s, China’s land use policies mainly focused on grain production due to the rapid population growth and rapid development of industry and agriculture, resulting in large-scale forest destruction and grassland reclamation in North China [1]. Since the Household Production Responsibility System (HPRS) began in the early 1980s, it has stimulated the rapid development of livestock breeding to meet the increasing demands of human population growth [2]. This sharp increase in livestock numbers placed heightened pressure on grassland utilization, and the subsequent overgrazing by livestock caused large-scale grassland degradation in China which, in turn, caused further overgrazing; this became a vicious circle [3,4,5]. This has even resulted in the desertification of areas with fragile environmental conditions and poor ecosystem structures, consequently leading to dust storms in North China [6,7]. Since the 1990s, the consequences of grassland degradation have led to an ever-growing interest in eco-compensation schemes to deal with environmental and agri-environmental issues in China [8,9,10]. In response to the ever-deteriorating condition of grasslands, the Chinese central government initiated the Returning Grazing Land to Grassland Program (RGLTGP) in the early 2000s which included the Prohibited Grazing Policy (PGP) in severe grassland degradation and ecologically fragile regions [11]. This policy is tasked with the dual goals of both ecosystem protection and poverty alleviation in China’s developing rural areas [8]. Consequently, in the initial years of the programs, the government offered feed grain subsidies or equivalent monetary subsidies [12]. Since the beginning of the 12th Five-Year Plan in 2011, a formalized set of grazing restrictions and compensatory payments was rolled out to eight pastoral provinces and autonomous regions, as well as the Xinjiang Production and Construction Corps, under the title of Grassland Ecosystem Subsidy and Award Scheme (GESAS). This scheme includes full grazing bans with compensation payments for severely degraded grasslands and reward payments for herders who operate within contracted stocking rates on grasslands that are in better condition [10]. The annual compensation payment from the central government to herders includes a subsidy for the grazing ban (112.5 CNY/ha/year in 2017; 6.6 CNY~1 USD in 2017); a subsidy for pastoralist’s production materials (500 CNY/household/year); and a grass seed subsidy 150 CNY/ha in 2017. The central government invests more than 15 billion CNY annually for grassland ecology subsidy and a large part of that is used for the forbidden grazing subsidy in the major grassland and pastoral areas in the eight provinces of China [13].

This paper mainly focused on the Grazing Prohibition Policy (PGP), for these participating areas are severe grassland degradation and ecologically fragile regions. Since this policy’s initiation in China, positive trends in ecological restoration have emerged [14,15], and land desertification has been reduced or reversed in some agro-pastoral transitional zones [7,16,17], although some scholars argue that grassland degradation is still a serious problem and that the existing programs need to be strengthened [18,19]. Such a policy has saliently influenced the peasant households’ income, livelihood strategies, and migration to the city thus affecting their life satisfaction. Zhang et al. [10] argues that the ongoing effectiveness of PES schemes on grassland conditions will depend on the extent to which herders adapt to the management practices which in turn will depend on whether farmers’ associate utility or satisfaction with such practices. The farmers are the main stakeholders in the scheme, and the long-lasting prospects of the policy will depend strongly on their behavior. Therefore, farmers who have a higher quality of life and policy satisfaction will be more supportive of policy implementation. For the policy to be successful, a good understanding of the determinants of PGP satisfaction, life satisfaction, and, particularly, of the relationship between PGP satisfaction and overall life satisfaction is required. Findings that elucidate the factors affecting PGP satisfaction and life satisfaction could help to identify specific strategies to simultaneously achieve ecosystem conservation and an improvement in farmers’ quality of life.

Previous studies have indicated that there are various factors that influence farmers’ satisfaction with the grassland ecological compensation policy. Wang and Liu (2017) [20] proposed that the factors affecting herders’ grassland ecological compensation policy satisfaction involved the herder’s education, number of livestock raised, changes in the weight of livestock, amount of subsidy received, the relative importance of the grassland environment and economic benefits, and the evaluation of social welfare. Chen (2013) [21] argues that the farmers who get subsidies are more satisfied with the ecological policy. The results of Hu et al. (2016) [22] show that there is a significantly positive correlation between ecological policy satisfaction and real income impact. Li et al. (2014) [23] suggest that farmers’ ethnic groups, amount of subsidy, forage expenditure, and self-reported subsidy standards have significant impact on ecological policy satisfaction. Zhang (2018) [24] holds that the herder’s education, number of livestock raised, household income, self-reported subsidy level, the influence of the ecological policy on the number of livestock raised, grassland environment as well as farmers’ living standards have significant effects on such policy satisfaction. These studies mainly focus on the factors affecting farmers’ satisfaction with the grassland ecological compensation policy including farmers’ demographics, received subsidy, and degree of the changes in the grassland [20,21,22,23,24]. However, the factors that affect PGP satisfaction may also play a role in life satisfaction. This study therefore seeks to explore the factors influencing PGP satisfaction and life satisfaction and contributes to previous studies by introducing the consideration of life satisfaction to the analysis of policy satisfaction.

## 2. Materials and Methods

### 2.1. Overview of the Study Area

**Figure 1:**
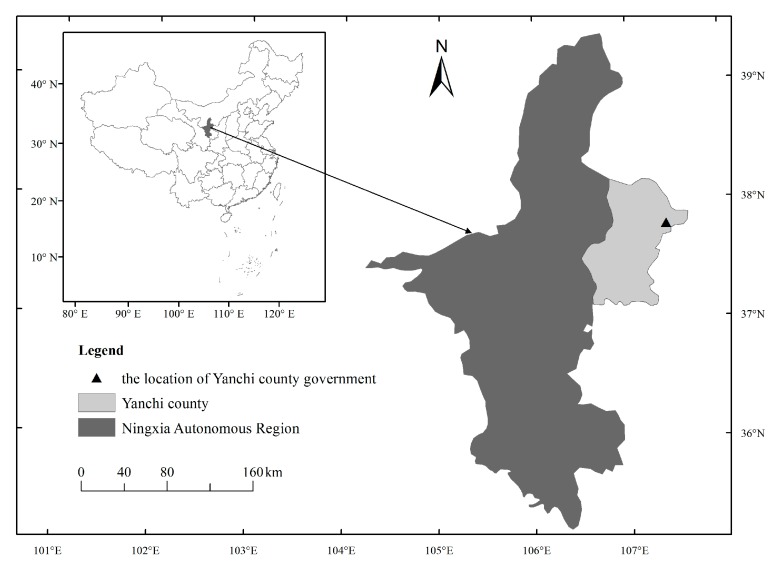
The study area.

There are eight pastoral provinces and autonomous regions that have implemented the grassland ecological compensation policy: Qinghai Province, Sichuan Province, Gansu Province, Yunnan Province, Ningxia Hui Autonomous Region, Tibet Autonomous Region, Inner Mongolia Autonomous Region, and Xinjiang Uygur Autonomous Region. We selected Yanchi County in Ningxia Hui Autonomous Region (Figure 1) as the research area, because this area is a typical transitional zone of topography, climate, vegetation, as well as farming and animal husbandry modes of production. This area is, additionally, an important ecological protection barrier for the middle reaches of the Yellow River and is a national key ecological function area [25]. In addition, “Ecological Yanchi” has been one of the three special place cards of this county. Through ecological propaganda, Yanchi County has greatly improved its popularity and has become a hotspot of scientific research. Thus, under the background of China’s cultural tradition that emphasizes harmony between human beings and nature [26], research in this area can provide reference for ecological construction in similar areas.

The total land area of this county is 8522 km^2^, and the grassland area is approximately 5570 km^2^, all of which participates in the PGP. The climate of Yanchi County is a typical temperate continental climate: dry, rainless, windy, and, therefore, dusty. The average annual precipitation is a mere 290 mm [27]. Grazing and livestock production was the main income source for farmers before the PGP, but the livelihood strategies have changed markedly since the PGP was implemented. Yanchi County, in the agro-pastoral transitional zone of North China, is ecologically fragile and was once a severe grassland degradation region. Prior to the policy, the desertification was very severe in this county and even once accounted for 52% of the total land area [28]. The whole county initiated the PGP in 2002; since this time, the area of desertification has significantly reduced and the intensity of desertification has reversed by a remarkable amount [29].

The rural per capita net income in Yanchi County was 9550 CNY in 2017. This has been increasing very rapidly, and the people in Yanchi County successfully overcame poverty in 2018 as did many other similar areas in Northwest China. In 2016, the rural population was 85.6 thousand, accounting for 55% of the total population of 155.7 thousand [29]. While Han people are in the majority, there are also over 4500 minority nationality people in Yanchi County accounting for 2.7% of the total population, most of whom are Hui people. Yanchi County has always been a place where agricultural and animal husbandry cultures interact with each other. The production of this area is deeply influenced by the two cultures that caused the coexistence of animal husbandry and agricultural production; thus, this area is known as “licorice town” and “Tan sheep town” [30].

### 2.2. Questionnaire Design and Data Collection

The dependent variables of this study were PGP satisfaction and overall life satisfaction; self-reported PGP satisfaction was measured using a five-point Likert scale question (1 = very dissatisfied to 5 = very satisfied), and the self-reported life satisfaction was measured using a 1–10 scale that ranged from “extremely dissatisfied” to “very satisfied” that is consistent with the World Values Survey (WVS) [31]. A substantial amount of literature has proved the reliability, validity, and comparability of the answers to such single subjective well-being (SWB) questions [32,33,34]. We focused here on the determinants of satisfaction, and measuring it using single items was feasible.

The potential explanatory factors in the questionnaire included: the (1) demographic characteristics of participants, (2) family economy, (3) policy perception, and (4) environmental perception. Table 1 lists the variables and questions used to explore the factors influencing policy satisfaction and life satisfaction. The previous studies showed that income, including absolute income and relative income, was associated with the respondents’ life satisfaction [35,36]. Thus, in the current study, we used the logarithm of household monthly income and the self-reported income level in the respondents’ village to represent the respondents’ absolute income and relative income, respectively. As has already been mentioned, previous studies have shown that the number of livestock raised has a significant effect on ecological policy satisfaction; however, only 34.4% of total respondents still raised sheep in Yanchi County according to this survey. We therefore decided to remove this variable. In addition, we used the variable of raising sheep (or not) to represent the farmers’ animal husbandry production mode due to the fact that raising sheep is the main mode of grassland animal husbandry in Yanchi County. This variable is affected by the PGP and may have a significant effect on PGP and life satisfaction. Previous studies showed that non-agricultural income, self-reported income, and expenses have an effect on respondents’ satisfaction [37,38]; thus, we added these two variables into the study.

Previous studies have shown that policy-related variables have an effect on satisfaction [23,24], so we used the self-reported influence of the PGP on household income, self-reported influence of the PGP on environment, and the self-reported subsidy level as the policy-related variables. The aim of the PGP is to improve the ecological environment; thus, the self-reported environmental quality and environmental satisfaction may have an effect on both PGP and life satisfaction. Finally, we also controlled for respondents’ personal demographic characteristics including age, gender, marital status, and education level.

Data for this study were obtained from a survey of rural households in Yanchi County undertaken in September 2018. We randomly selected four or five villages from a list of villages in each town of the eight townships as the sample area. A total of 36 villages were selected as survey areas, and four to ten respondents were randomly selected from each village when we entered the village according to the size of the village. Because many rural residents are illiterate, face-to-face household interviews were carried out in our survey. One household was surveyed only with a questionnaire by someone who was familiar with their family situation. A total of 253 complete questionnaires were collected from this county.

### 2.3. Model Selection

Because the dependent variables (i.e., PGP satisfaction and overall life satisfaction) used in this study were ordinal variables, we used the ordered probit model to investigate the effects of various determinants on PGP and life satisfaction:

(1) $$y_{i}^{*}=\;\alpha \;+\;\beta_{i}X_{i}\;+\;\varepsilon$$

where $y_{i}^{*}$ denotes individual PGP satisfaction or life satisfaction, and the vector *X* includes the explanatory variables in the study.

In this study, PGP satisfaction may be correlated with the error term in the life satisfaction regression, as some people may be more easily satisfied with both their life and the policy. Thus, the stochastic component of PGP satisfaction may be correlated with the stochastic element of life satisfaction. To resolve this potential endogeneity problem, we needed to remove the stochastic element from PGP satisfaction by generating the predicted values from PGP satisfaction equation. As in the Ramsey regression equation specification error test (RESET), the squared and cubed terms of the fitted value of PGP satisfaction from Equation (1) were generated and added to the expanded Equation (2):

(2) $${\hat{y}}_{i}^{*}=\;\alpha \;+\;\beta_{i}X_{i}\;+\delta_{1}{\hat{y}}^{2}+\delta_{2}{\hat{y}}^{3}\;+\varepsilon$$

where $\hat{y}$ denotes the fitted value of PGP satisfaction from Equation (1), ${\hat{y}}_{i}^{*}$ denotes the predicted value of PGP satisfaction that was used in the life satisfaction regression, and then the regression was used to compare with the regression used for the original PGP satisfaction [39].

Additionally, robustness checks were employed firstly by omitting some variables with high correlation coefficients and, secondly, by transforming the dependent variable into a 0–1 binary variable, then estimating the regression by a probit model. In order to conduct the ordered probit model, Stata 15.0 (StataCorp, Texas, TX, USA) was used in the current study.

## 3. Results

### 3.1. Descriptive Results

In Table 2, we show the distribution of PGP satisfaction as well as the average PGP satisfaction and life satisfaction for different individual characteristics, family economy, and livelihood strategies of animal husbandry. The results show that there were only 17.8% respondents who were unsatisfied or neutral regarding the PGP; this is similar to previous results [21,23,24]. Thus, most farmers were satisfied with the PGP and approved of this policy in their area.

The PGP changed farmers’ livelihoods markedly. Prior to the implementation of this policy, every family raised sheep in Yanchi County. According to our survey, 87 families currently raise sheep, accounting for only 34.4% of total respondents. The independent sample *t*-test (*t* = 2.296, *p* < 0.05) shows that there was a significant difference in PGP satisfaction between the farmer’ who raised sheep and those who did not raise sheep; the average PGP satisfaction of farmers raising sheep was less than that of farmers not raising sheep, but there was no significant difference in life satisfaction (*t* = −0.117, *p* > 0.1). Therefore, to analyze the different effects of livelihood strategies on PGP satisfaction among groups, we divided the full sample by grassland animal husbandry livelihood strategies (those who raised sheep and those who did not) in the regression analysis that follows.

### 3.2. Effects on PGP Satisfaction

As shown in Table 3, for the full sample, the results of the ordered probit model indicate that the self-reported influence of the PGP on household income, subsidy level, environmental satisfaction, self-reported influence of the PGP on environment, and self-reported environmental quality had significantly positive effects on PGP satisfaction. However, farmers’ economic status (including absolute income and self-reported relative income) had no significant effect on their PGP satisfaction.

In addition, raising sheep had a significantly negative impact on PGP satisfaction. Table 4 shows the marginal effect of raising sheep on PGP satisfaction via the ordered probit model, based on column (1) in Table 3. Specifically, from the estimation results in Table 4, compared with farmers who do not raise sheep, the proportion of farmers raising sheep who were “very satisfied” with the PGP was 12.9% lower, the proportion for “satisfied” was 5.0% higher, the proportion for “neutral” was 3.8% higher, the proportion for “dissatisfied” was 2.0% higher, and the proportion for “very dissatisfied” was 2.2% higher. The results indicate that grassland-related livestock breeding had a significantly adverse impact on farmers’ policy satisfaction. For the farmers who raise sheep, grassland-related livestock are the major source of family income, and implementation of the PGP increased the costs of raising sheep. In order not to affect their income, many of them will graze illegally at night. Our survey shows that, for the farmers who raised sheep, 61% grazed their sheep at night. Long-term illegal grazing at night is detrimental to both their physical and mental well-being, and thus impacts their policy satisfaction.

To assess robustness, the independent variables of the log monthly household income and self-reported environmental quality were excluded, and the results were similar to the original estimations. A second robustness check was performed by a binary variable probit regression, which resulted in the effect of raising sheep and the subsidy level being no longer significant. Of course, changing a dependent variable from an ordered to a binary one would entail a loss of information [39], and this information is important.

As shown in Table 3, the results among the samples of raising sheep and not raising sheep were similar. For the two groups, the farmers who had higher environmental satisfaction and considered that the PGP improved their income and environment enjoyed a higher PGP satisfaction.

### 3.3. Effects on Overall Life Satisfaction

As Table 5 shows, marital status, environmental satisfaction, self-reported influence of the PGP on income, self-reported income level in the respondents’ village, and self-reported income and expenditure had significantly positive effects on overall life satisfaction. However, raising sheep, subsidy level, self-reported influence of the PGP on environment, and self-reported environmental quality all had an insignificant impact on life satisfaction. Meanwhile, both of the robustness checks were in accordance with the original ordered probit (see Table 5). When we included PGP satisfaction as an explanatory variable, it had a significantly positive effect on overall life satisfaction. The effects of other variables were in accordance with the results of not including the PGP satisfaction. Thus, we can conclude that the farmers’ policy perception, environmental perception, and livelihood strategy affect their PGP satisfaction more than their overall life satisfaction. Even so, environmental satisfaction and the self-reported influence of the PGP on income were powerful in explaining both PGP satisfaction and life satisfaction.

### 3.4. Effects on Life Satisfaction Using the Predicted Value of PGP Satisfaction

As mentioned in Section 2.3., PGP satisfaction may be correlated with the error term in the overall satisfaction regression. To resolve the potential endogeneity problem, the predicted values of PGP satisfaction from the expanded Equation (2) were used in the life satisfaction regression.

Table 6 shows the comparisons between the regression using predicted values of PGP satisfaction and the original regression from Table 5; the two regressions showed quite similar results. The PGP satisfaction, marital status, self-reported income level in the respondents’ village, and self-reported income and expenditure played an important role in determining people’s life satisfaction. The modified regression suggests that self-reported influence of the PGP on household income and environmental satisfaction no longer significantly influenced life satisfaction. That may be because the impacts of these two variables were already captured by the predicted explanatory variable of PGP satisfaction. In any case, other regression results have shown their importance.

## 4. Discussion

### 4.1. The Effects of PGP Satisfaction on Life Satisfaction

The results of this study show that PGP satisfaction has a significantly positive effect on overall life satisfaction. The bottom-up spillover theory of subjective well-being (SWB) recognizes that satisfaction with one’s life is mostly determined by satisfaction with a variety of life domains [32,40,41,42,43]. Thus, it is argued here that the effect of PGP satisfaction on life satisfaction is a type of bottom-up spillover effect. The farmers’ overall life satisfaction could be significantly affected by PGP satisfaction which indicates the importance of the PGP in the daily lives of the farmers who participate in the policy.

### 4.2. The Effects of Livelihood Strategies

Since the initiation of the PGP, farmers’ livelihood strategies have been severely impacted. Prior to the emergence of the policy, every family raised sheep, leading to a *tragedy of the commons*. Every farmer obtained the full benefit of placing sheep on the common grasslands while suffering only a small share of the cost to the community as a whole [44]. The huge livelihood pressure made overgrazing and grassland degradation inevitable. However, after 16 years of the policy’s implementation, the results of our survey show that there were only 34.4% of total respondents who still raised sheep in Yanchi County. The PGP forced farmers to feed their sheep in the barn therefore buying more feed which increased the cost of raising livestock. This spurred farmers to increase the breeding scale or turn to other strategies to earn an income. Additionally, an ageing population has also compelled older farmers to retire from raising sheep; according to our survey, the average age of the farmers who did not raise sheep was 59.1, while the average age of the farmers who did raise sheep was 53.6. Previous studies showed that economic development promotes rapid urbanization which in turn stimulates massive rural-to-urban migrations [7]. Younger people, especially those born post-1980 and post-1990, prefer urban life and engage in off-farm work. In 2016, nearly 170 million peasant workers left their home villages and moved to cities, most of them young and better educated [45]. All of this promotes a reduction in the number of farmers who raise sheep. It is expected that the number of farmers who raise sheep will be further reduced as they get older, and whether it is necessary to implement such a strict policy in the future still needs further study in these areas.

**Figure 2:**
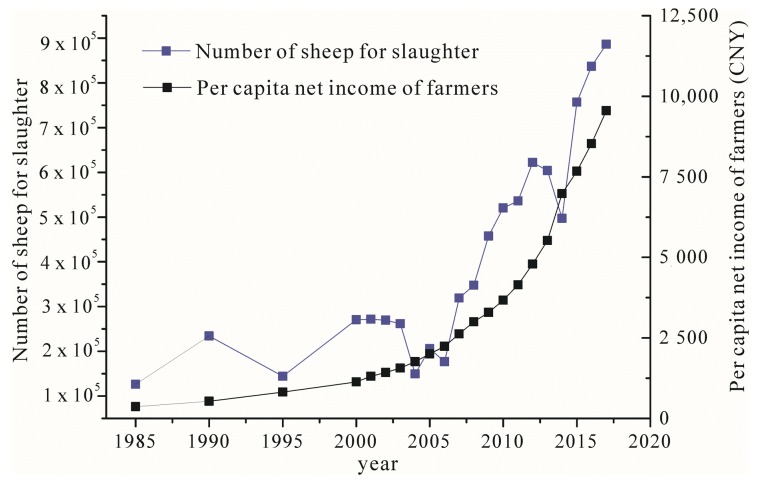
The change in the number of sheep for slaughter and per capita net income of farmers in Yanchi County.

The aims of the PGP are to improve the ecological environment without damaging the living standards of farmers. If animal husbandry productivity can be sufficiently enhanced by more intensive feeding or more advanced technology on limited grasslands, the PGP might not inevitably lead to declining farm income [10]. The data from the Yanchi County Statistical Yearbook 2017 show that the per capita net income of farmers in Yanchi County has continued to grow since the launch of the PGP (Figure 2). However, the number of sheep for slaughter decreased in the first three years following the PGP’s implementation, after which there was a quick rebound; the number of sheep for slaughter increased from approximately 269 thousand in 2002, to more than 886 thousand in 2017 (Figure 2). After the PGP, the farmers who raised sheep enlarged their sheep breeding scale in order to buffer the impacts of the PGP on their income. According to the results of our survey, the average number of sheep raised per household in Yanchi County was 113 in 2018, compared to 54 at the beginning of the PGP [46]. The PGP compels farmers to turn to more intensive feeding and more advanced technology, and, coupled with the various subsidies from the government, the PGP will alleviate poverty. Additionally, there are sheep breeding enterprises that conduct intensive livestock feeding which promoted the capitalization of sheep farming. Government policies are also promoting the development of animal husbandry; in 2017, there was a 30 CNY subsidy for slaughtering one sheep. Intensive livestock feeding, development of breeding technology, and the encouragement of relevant government policies all have promoted the rapid growth of animal husbandry in Yanchi County after the initial impact of the PGP.

To accommodate and encourage a transition to more intensive feeding, future versions of the Grassland Ecosystem Subsidy and Award Scheme (GESAS) and associated grassland policies will need to reinforce investments and subsidies for the construction of artificial grasslands, forage bases, and livestock sheds in the agro-pastoral ecotones in North China that are similar to Yanchi County. Additionally, the farmers who do not raise sheep may grow cash crops or work off-farm for income. This could all enhance the farmers’ quality of life and, therefore, their life satisfaction. Previous studies have indicated that the SWB of the people who migrate to cities is mainly affected by issues of social justice and life stress. Even when rural migrant workers work and pay taxes in cities, the national household registration system still identifies them as rural people. Thus, they are not entitled to social welfare in the cities where they live [47,48]. However, for those who are not engaged in grassland animal husbandry, their PGP satisfaction and life satisfaction do not affect the primary purpose of the policy implementation to improve the grassland ecological environment. But the policy could enhance their SWB to boost the sustainable development of society.

### 4.3. The Role of Relative Income

Our results show that the self-reported income level in the respondents’ village had a significantly positive impact on life satisfaction. This means that farmers who have higher income level perceptions will have higher life satisfaction. However, the impacts of the absolute income on the PGP satisfaction and life satisfaction were not significant. The SWB is strongly affected by relative income which is consistent with other studies [49,50,51,52,53]. Due to the fact of hedonic adaption, social comparison, and aspirations improving with absolute income, the absolute income level had little effect on SWB, and status relative to others becomes very important [54,55,56]. This may indicate that the visual presence of income equity affects residents’ satisfaction in developing countries [57].

### 4.4. Limitations of the Current Study

There are several limitations in this article. Firstly, this study was conducted in a local area; a comparative study of other similar regions should be conducted in the future. Secondly, we only collected one year of survey data; therefore, our results would be strengthened by more years of data for comparison in the future. Moreover, the respondents of our survey were chosen to be those who are familiar with their family situations, and one household was surveyed only with a questionnaire which resulted in all of the respondents being adult. It may seem that children are also potential respondents, and we believe children’s satisfaction should be considered in future studies. In addition, previous studies have argued that China’s land reform could only succeed if the clarification of property rights is conducted, but the grassland area per capita in Yanchi County is relatively small and grazing is forbidden, so the effectiveness of the clarification of property rights in regions similar to Yanchi County still needs further study [58]. We do, however, expect an increase in PGP satisfaction and life satisfaction with the current trends of improvement in the ecological environment and social welfare.

## 5. Conclusions

This study investigated the factors influencing respondents’ PGP satisfaction and life satisfaction in rural Northwest China. Our empirical results suggest that raising sheep, self-reported influence of the PGP on income and environment, subsidy level, environmental satisfaction, and self-reported perception of environmental quality significantly affect PGP satisfaction. Thus, we can conclude that farmers’ policy perceptions, environmental perceptions, and livelihood strategies significantly impact on their PGP satisfaction. Further, PGP satisfaction, marital status, environmental satisfaction, self-reported influence of the PGP on income, self-reported income level, and self-reported income and expenditure have significantly positive effects on overall life satisfaction. The PGP could be used not only to improve the grassland ecological environment, but also to actively promote the farmers’ life satisfaction by improving their PGP satisfaction. China’s policy is the emphasis on a people-centered approach, so the government should improve satisfaction in the PGP in order to enhance life satisfaction and promote social stability.

The improvement of PGP satisfaction and life satisfaction does not mean that the PGP is being implemented well [22], but the higher satisfaction could make farmers more supportive and willing to participate in such policy. Relevant policies, infrastructure, and social security to promote grassland restoration and quality of life could make such a policy easier to regulate. The PGP areas in China are generally poor, and poverty eradication and ecosystem conservation are among the major goals being targeted by the 2030 Agenda for Sustainable Development of the United Nations [59,60]. Approaches that improve farmers’ satisfaction could reduce poverty simultaneously. Therefore, policymakers and stakeholders should work to improve farmers’ PGP satisfaction and life satisfaction according to our relevant research results which could promote better implementation of such programs, social stability, and sustainable development.

## Figures and Tables

**Table 1:** Description of variables in the model (*N* = 253).

Influencing Factors	Variables	Variable Assignment	Mean (SD)
Subjective well-being	Life satisfaction	1 = “Extremely dissatisfied” to 10 = “very satisfied”	7.70 (2.02)
	PGP satisfaction	1 = “very dissatisfied” to 5 = “very satisfied”	4.07 (0.92)
Personal characteristics	Age	1 = 44 years old and below, 2 = 45–59 years old, 3 = 60 years old and above	2.32 (0.70)
	Male	1 = male, 0 = female	0.54 (0.50)
	Married	1 = married, 0 = not married and other	0.91 (0.29)
	Education level	1 = uneducated, 2 = primary school, 3 = middle school, 4 = senior high, 5 = college and above	2.06 (0.95)
Family economy	Log monthly household income		3.44 (0.45)
	Income level in your village	1 = “very low” to 5 = “very high”	2.57 (0.84)
	Raising sheep	1 = raise sheep, 0 = does not raise sheep	0.34 (0.48)
	Non-agricultural monthly income	1 = less than 2000 CNY, 2 = 2000–5999 CNY, 3 = 6000 CNY and above	1.41 (0.60)
	Income and expenses	1 = “have debt” to 4 = “save money”	2.76 (0.91)
Policy perception	Influence of PGP on income	1 = “strongly worsen” to 5 = “strongly improve”	2.72 (0.87)
	Subsidy level	1 = “very low” to 3 = “reasonable and high”	2.44 (0.76)
	Influence of PGP on environment	1 = “strongly worsen” to 5 = “strongly improve”	4.84 (0.46)
Environmental perception	Satisfaction with environment	1 = “very dissatisfied” to 5 = “very satisfied”	4.22 (0.70)
	Perceived environmental quality	1 = “very poor” to 5 = “very good”	4.31 (0.63)

**Table 2:** Demographics of the participants and their satisfaction levels (*N* = 253).

Parameters	Distribution (Percentage)	PGP Satisfaction	Life Satisfaction
		Mean (SD)	Mean (SD)
*PGP satisfaction*			
Unsatisfied or neutral	45 (17.8%)	-	-
Relatively satisfied	122 (48.2%)	-	-
Very satisfied	86 (40.0%)	-	-
*Gender*			
Male	136 (54%)	4.07 (0.98)	7.81 (1.96)
Female	117 (46%)	4.06 (0.85)	7.58 (2.09)
*Age*			
< 45	35 (14%)	4.00 (0.94)	7.40 (1.88)
45–59	102 (40%)	3.95 (0.88)	7.60 (2.07)
> 59	116 (46%)	4.19 (0.94)	7.89 (2.01)
*Education*			
Lower than primary education	83 (33%)	4.16 (1.01)	7.49 (2.03)
Primary education	94 (37%)	3.96 (0.88)	7.81 (2.09)
Middle school	59 (23%)	4.17 (0.72)	7.83 (2.04)
Senior high	13 (5%)	3.92 (1.12)	7.46 (1.27)
Higher education	4 (2%)	3.75 (1.89)	8.50 (1.92)
*Household income (CNY/Month)*			
< 2000	90 (36%)	4.17 (0.88)	7.68 (2.18)
2000–5999	99 (39%)	3.92 (0.98)	7.62 (1.94)
> 5999	64 (25%)	4.16 (0.88)	7.87 (1.92)
*Livelihood strategies*			
Raising sheep	87 (34%)	3.89 (1.06)	7.72 (1.87)
Not raising sheep	166 (66%)	4.16 (0.83)	7.69 (2.10)
*t* (d*f* = 251)		2.296	−0.117
*p* (two-tail)		*p* < 0.05	*p* > 0.1

**Table 3:** Ordered probit regression results. Dependent variable: PGP satisfaction.

Variables	Total Sample	Robustness Checks		By Groups	
	Total	Drop Independent Variable	Binary Dependent Variable (0–1)	Raise Sheep	Do Not Raise Sheep
	Coefficient	Coefficient	Coefficient	Coefficient	Coefficient
Age	−0.061 (−0.46)	−0.026 (−0.21)	−0.193 (−0.94)	−0.073 (−0.53)	−0.032 (−0.20)
Male	0.088 (0.56)	0.101 (0.64)	0.001 (0.00)	0.288 (1.00)	−0.031 (−0.15)
Married	−0.000 (−0.00)	−0.026 (−0.10)	0.307 (0.77)	−0.078 (−0.15)	−0.026 (−0.08)
Education	−0.029 (−0.30)	−0.054 (−0.56)	0.010 (0.06)	−0.040 (−0.24)	0.007 (0.05)
Log monthly household income	−0.176 (−0.73)	-	−0.505 (−1.39)	−0.559 (−1.20)	−0.008 (−0.03)
Non-agricultural income	0.150 (0.96)	0.118 (0.91)	0.440 (1.70)	0.383 (1.58)	−0.032 (−0.15)
Raising sheep	−0.452 ** (−2.56)	−0.482 *** (−3.02)	−0.379 (−1.43)	-	-
Income level in your village	0.026 (0.25)	0.047 (0.48)	−0.166 (−1.02)	0.114 (0.52)	0.018 (0.16)
Income and expenditure	0.067 (0.76)	0.040 (0.46)	0.136 (1.04)	0.146 (1.00)	0.035 (0.30)
Influence of PGP on income	0.303 *** (3.33)	0.279 *** (3.10)	0.308 ** (2.34)	0.438 *** (2.68)	0.255 ** (1.84)
Subsidy level	0.175 * (1.69)	0.205 ** (2.00)	0.194 (1.30)	0.200 (0.97)	0.115 (0.90)
Influence of PGP on environment	0.540 *** (3.20)	0.534 *** (3.18)	1.062 *** (4.36)	0.731 ** (2.07)	0.477 ** (2.34)
Satisfaction with environment	0.543 *** (3.36)	0.763 *** (6.28)	0.628 *** (2.76)	0.629 ** (2.34)	0.582 ** (2.43)
Perceived environmental quality	0.360 ** (2.04)	-	−0.029 (−0.12)	0.293 (1.19)	0.395 (1.45)
_cons			−6.329 (−3.88)		
Log likelihood	−239.538	−241.914	−80.594	−89.986	−145.880
LR chi^2^	123.38	118.62	75.69	44.31	79.85
Prob > chi^2^	0.000	0.000	0.000	0.000	0.000
Pseudo *R*^2^	0.205	0.197	0.320	0.198	0.215

Note: Parentheses denotes the z-statistics of the respective coefficients, * *p* < 0.1, ** *p* < 0.05, *** *p* < 0.01.

**Table 4:** Marginal effect of raising sheep on PGP satisfaction.

PGP Satisfaction	Order Probit (Marginal Effects)
Very dissatisfied	0.022 ** (2.23)
Dissatisfied	0.020 ** (2.09)
Neutral	0.038 ** (2.36)
Satisfied	0.050 ** (2.44)
Very satisfied	−0.129 *** (−2.62)
Other variables	Yes

Note: Parentheses denotes the *z*-statistics of the respective coefficients, ** *p* < 0.05, *** *p* < 0.01.

**Table 5:** Ordered probit regression results. Dependent variable: life satisfaction.

Variables	Total Sample			
	Total	Including PGP Satisfaction	Robustness Checks	
			Drop Independent Variable	Binary Dependent Variable (0–1)
	Coefficient	Coefficient	Coefficient	Coefficient
PGP satisfaction	-	0.195 ** (2.18)	-	-
Age band	0.044 (0.38)	0.058 (0.49)	0.048 (0.43)	0.139 (0.94)
Male	0.128 (0.88)	0.120 (0.83)	0.123 (0.85)	0.113 (0.62)
Married	0.533 ** (2.32)	0.524 ** (2.27)	0.536 ** (2.33)	0.445 ** (1.50)
Education	0.007 (0.07)	0.013 (0.15)	0.010 (0.11)	0.061 (0.53)
Log monthly household income	−0.035 (−0.16)	0.011 (−0.05)	-	0.047 (0.17)
Non-agricultural income	0.019 (0.13)	0.003 (0.03)	−0.002 (−0.02)	−0.024 (−0.14)
Raising sheep	−0.025 (−0.16)	0.037 (0.23)	−0.043 (−0.30)	−0.014 (−0.07)
Income level in your village	0.216 ** (2.37)	0.211 ** (2.32)	0.208 ** (2.30)	0.136 (1.18)
Income and expenditure	0.244 *** (2.97)	0.241 *** (2.93)	0.248 *** (3.04)	0.263 ** (2.50)
Influence of PGP on income	0.233 *** (2.80)	0.197 ** (2.33)	0.235 *** (2.86)	0.281 *** (2.61)
Subsidy level	−0.108 (−1.12)	−0.134 (−1.37)	−0.116 (−1.22)	−0.107 (−0.87)
Influence of PGP on environment	0.143 (0.91)	0.037 (0.22)	0.139 (0.89)	0.433 (1.92)
Satisfaction with environment	0.436 *** (2.79)	0.372 ** (2.40)	0.356 *** (3.22)	0.411 ** (2.01)
Perceived environmental quality	−0.111 (−0.68)	−0.137 (−0.83)	-	−0.161 (−0.75)
_cons				−5.632 (−3.97)
Log likelihood	−437.858	−435.486	−438.098	−150.833
LR chi^2^	62.28	67.02	61.80	46.97
Prob > chi^2^	0.000	0.000	0.000	0.000
Pseudo *R*^2^	0.066	0.072	0.066	0.135

Note: Parentheses denotes the *z*-statistics of the respective coefficients, ** *p* < 0.05, *** *p* < 0.01.

**Table 6:** Comparisons of life satisfaction regressions.

Variables	Total Sample	
	Regression Using the Predicted Value of PGP Satisfaction	Original Regression Including PGP Satisfaction
	Coefficient	Coefficient (Table 5)
PGP satisfaction	0.337 * (1.80)	0.195 ** (2.18)
Age band	0.048 (0.41)	0.058 (0.49)
Male	0.109 (0.75)	0.120 (0.83)
Married	0.613 *** (2.61)	0.524 ** (2.27)
Education	0.014 (0.16)	0.013 (0.15)
Log monthly household income	−0.010 (−0.05)	0.011 (−0.05)
Non-agricultural income	0.002 (0.01)	0.003 (0.03)
Raising sheep	0.145 (0.78)	0.037 (0.23)
Income level in your village	0.197 ** (2.15)	0.211 ** (2.32)
Income and expenditure	0.236 *** (2.87)	0.241 *** (2.93)
Influence of PGP on income	0.125 (1.22)	0.197 ** (2.33)
Subsidy level	−0.197 * (−1.82)	−0.134 (−1.37)
Influence of PGP on environment	0.213 (1.31)	0.037 (0.22)
Satisfaction with environment	0.130 (0.58)	0.372 ** (2.40)
Perceived environmental quality	−0.176 (−1.05)	−0.137 (−0.83)
_cons		
Log likelihood	−436.233	−435.486
LR chi^2^	63.53	67.02
Prob > chi^2^	0.000	0.000
Pseudo *R*^2^	0.070	0.072

Note: Parentheses denotes the z-statistics of the respective coefficients, * *p* < 0.1, ** *p* < 0.05, *** *p* < 0.01.
